# A Simple Nomogram for Predicting Stroke-Associated Pneumonia in Patients with Acute Ischemic Stroke

**DOI:** 10.3390/healthcare11233015

**Published:** 2023-11-22

**Authors:** Youn-Jung Lee, Hee Jung Jang

**Affiliations:** 1 Department of Nursing, Hallym Polytechnic University, Chuncheon 24210, Republic of Korea; sm7179@hanmail.net; 2 School of Nursing, Research Institute of Nursing Science, Hallym University, Chuncheon 24252, Republic of Korea

**Keywords:** stroke, pneumonia, logistic models, nomograms, clinical nursing research

## Abstract

The purpose of this study was to develop a prediction model for stroke-associated pneumonia (SAP) based on risk factors for SAP and to suggest nursing interventions to prevent SAP. In addition, a nomogram was developed to enhance its utility in nursing practice. The retrospective cohort study included 551 patients hospitalized for acute ischemic stroke at a university hospital in South Korea. Data were collected through a structured questionnaire and a review of the electronic medical record (EMR). In the development of a predictive model for SAP, multivariate logistic regression analysis showed that independent risk factors for SAP were age ≥ 65 years, National Institute of Health Stroke Scale (NIHSS) score ≥ 7, nasogastric tube feeding, and C-reactive protein (CRP) ≥ 5.0 mg/dL. The logit model was used to construct the SAP prediction nomogram, and the area under the curve (AUC) of the nomogram was 0.94. Furthermore, the slope of the calibration plot was close to the 45-degree line, indicating that the developed nomogram may be useful for predicting SAP. It is necessary to monitor the age, NIHSS score, nasogastric tube feeding status, and CRP level of stroke patients and identify high-risk groups using the developed nomogram to provide active nursing interventions to prevent SAP.

## 1. Introduction

Stroke is a disease that occurs when a blood vessel in the brain is blocked or ruptured, causing damage to brain cells that receive blood through blood vessels, resulting in various neurological deficits such as movement disorders, sensory disorders, and dysarthria [1]. According to the WHO’s 2019 global cause of death statistics, stroke accounted for approximately 6 million deaths, ranking second only to cardiovascular disease among the top 10 causes of death [2]. Even if a person survives, stroke can cause complications such as hemiparesis, hemiparesis, sensory impairment, speech impairment, dysphagia, vision and visual field impairment, and loss of consciousness, depending on the lesion in the brain. There are also various internal complications such as heart disease, pneumonia, pressure ulcers, and deep vein thrombosis [1]. Therefore, the management of acute stroke patients is important, and patients with ischemic stroke can be treated with thrombolysis within 4–5 h of symptom onset, and patients with ischemic stroke with intracranial large artery occlusion can sometimes be treated with thrombectomy within 24 h. Patients with milder symptoms may have a delayed time from symptom onset to hospitalization.

Pneumonia that occurs within 7 days of hospitalization in stroke patients is known as stroke-associated pneumonia (SAP), which is one of the most common complications of stroke and affects around 20% of acute stroke patients [3]. SAP causes hypoxia of the brain cells, which leads to a decrease in overall brain function. It is also associated with a three-fold increase in mortality, an average of 7 additional days of hospitalization, and increased healthcare costs [4]. The AHA/ASA recognizes the importance of proactively preventing SAP in stroke patients to ensure rapid neurological recovery and a return to normal activities [5].

The need for SAP prevention has prompted studies on risk factors and predictive models. Generally, known risk factors include advanced age, dysphagia, and a high National Institute of Health Stroke Scale (NIHSS) score; however, there is no established risk factor, and each prediction model analyzes different risk factors and has different diagnostic criteria [6].

In order to improve the accuracy of prediction models, follow-up studies are needed to change or establish the risk factors or diagnostic criteria of a disease [7]. Recently, studies have shown that the neutrophil-to-lymphocyte ratio (NLR) may be a risk factor for SAP [8,9], and obesity has also been identified as a risk factor [10]. The NLR is generally regarded as a poor prognostic factor for pneumonia; however, there is a lack of research on its role as a predictor and its association with SAP. The relationship between obesity and pneumonia is also not well established, with different studies reporting different results. In 2015, the PISCES group proposed new diagnostic criteria for SAP and recommended that they be used uniformly [11]. The difference with previous diagnostic criteria is that while Mann et al.’s diagnostic criteria [12] include abnormalities in culture results, the PISCES group believes that culture testing is not necessary to diagnose SAP because SAP does not imply a pathophysiologic or microbiologic etiology. In addition, because pneumonia occurs most frequently within the first week of stroke onset, the PISCES group suggested using the CDC’s diagnostic criteria [13] for pneumonia but defining pneumonia occurring within 7 days of stroke onset as SAP and pneumonia occurring after 7 days as hospital-acquired pneumonia (HAP). This would reduce the likelihood of misdiagnosing HAP as SAP. However, there are still no predictive model studies that include the NLR and obesity as risk factors and no studies based on the new diagnostic criteria; thus, further research is needed.

Previous prediction models have used logistic regression models or risk scores to predict risk; however, logistic regression models are restricted to the use of complex mathematical formulas, and risk scores are easy to calculate but have low prediction accuracy [14]. Recently, research on risk prediction using nomograms, which compensate for these limitations, has been actively conducted. A nomogram can easily calculate the risk of occurrence of an outcome variable by utilizing a pictorial method, and it has the advantages of easy identification of high-risk groups and high prediction accuracy [14], so it is widely used in actual clinical practice, such as predicting the prognosis of cancer patients [15,16] or predicting the occurrence of diseases [17,18]. To our best knowledge, there are two studies that have used nomograms to predict SAP in patients with acute ischemic stroke, but one is based on CDC diagnostic criteria [19] and the other only includes patients with type 2 diabetes [20].

Therefore, this study aimed to develop a prediction model based on the new SAP diagnostic criteria for acute ischemic stroke patients and to suggest nursing interventions for SAP prevention. In addition, a nomogram was developed for possible utilization in nursing practice.

## 2. Materials and Methods

### 2.1. Study Design and Subjects

This study was a case–control study to develop a SAP prediction model and a nomogram based on risk factors for SAP and to evaluate their validity.

The subjects of this study were acute ischemic stroke patients aged 19 years or older and within 7 days of symptom onset who were hospitalized at a university hospital in South Korea from 1 January 2020 to 31 December 2021.

Exclusion criteria were patients who did not visit the hospital within 7 days of stroke onset, patients diagnosed as having pneumonia at the time of admission, patients who developed pneumonia after 7 days of admission, and patients with missing survey information in the medical records.

The sample size was determined by logistic regression analysis using the G*Power 3.1.9.7 (Heinrich-Heine-University, Dϋsseldorf, Germany). Based on previous research [21], an odds ratio of 1.8 was identified for advanced age, which is a common risk factor for SAP [22], so we applied an odds ratio of 1.8. The incidence of SAP was set to 10% based on previous research [23]. With a significance level (α) of 0.05 and power (1-β) of 0.90, the total number of subjects required was 491. With a 2:1 ratio of development to validation [24], 327 development subjects and 164 validation subjects were required.

### 2.2. Research Tools

The research tool was a structured questionnaire, and the risk factors used in this study included the main factors related to SAP indicated in previous meta-analysis studies [14,25], which were categorized into sociodemographic characteristics, clinical parameters, pre-existing comorbidities, and laboratory parameters based on the categories of a previous study [19]. The questionnaire was reviewed by a neurologist, an internist, a professor of adult nursing, and a nurse with more than 15 years of clinical experience.

Sociodemographic characteristics consisted of four factors: age, sex, smoking history, and alcohol consumption. Clinical characteristics consisted of nine factors: NIHSS, modified Rankin scale (mRS), dysarthria, dysphagia, Oxfordshire Community Stroke Project (OCSP) classification, body mass index (BMI), nasogastric tube feeding, ventilator use, and proton pump inhibitor (PPI) administration. Pre-existing comorbidities included previous stroke, hypertension, diabetes, chronic obstructive pulmonary disease, atrial fibrillation, congestive heart failure, ischemic heart disease, and chronic renal failure (8 factors). Laboratory parameters comprised 7 factors: serum albumin, white blood cells (WBC), C-reactive protein (CRP), glucose, NLR, blood urea nitrogen (BUN), and creatinine (Cr).

SAP was diagnosed according to the SAP diagnostic criteria defined by the PISCES group [11]. The diagnostic criteria were as follows: within 7 days of stroke onset, (1) meeting at least one of the following criteria: fever (>38.0 °C), leukopenia (<4000/mm^3^) or leukocytosis (≥12,000/mm^3^), and altered mental status (≥70 years of age), (2) with concomitant respiratory symptoms and/or signs, and (3) a positive chest radiograph.

### 2.3. Data Collection

The period of data collection for this study was from February to May 2022. The information collected using the questionnaire was reviewed through examining admission records, progress notes, discharge records, nursing records, consultation records, blood test results, and chest radiographs from the electronic medical record (EMR). The laboratory parameters were collected from tests performed at admission, and pre-existing comorbidities were collected from diagnoses recorded in the admission records, progress notes, discharge records, and consultation records. We also collected clinical parameters such as nasogastric tubes at the time of admission. However, in the case of PPIs, we set patients as PPI-treated if they had received PPIs during hospitalization based on previous studies [26]. Dysphagia was diagnosed at admission using the Standardized Swallowing Assessment (SSA) [27].

### 2.4. Data Analysis

Data analysis was performed using the IBM SPSS Statistics 28.0 statistical program (IBM Corp. Armonk, NY, USA) and R ver. 4.1.2 (R Foundation for Statistical Computing, Vienna, Austria).

The sociodemographic characteristics, clinical parameters, pre-existing comorbidities, and laboratory parameters of the study population are presented as frequencies and percentages or means and standard deviations. Comparisons of sociodemographic characteristics, clinical parameters, pre-existing comorbidities, and laboratory parameters between SAP groups and non-SAP groups were performed using chi-square tests for categorical variables and independent *t*-tests for continuous variables. For variables not normally distributed, the Mann–Whitney U test was used.

Multivariate logistic regression was used to identify risk factors that were significantly different between the SAP and non-SAP groups and to describe their association in a single model. Model fit was assessed by Nagelkerke R^2^ and the Hosmer–Lemeshow goodness-of-fit (GOF) test to determine the predicted probability of pneumonia for each subject. C-statistics were calculated using the SAP probability calculated from the model developed by logistic regression. Using Youden’s index on the ROC curve, we determined the value at which the sum of sensitivity and specificity is maximized as the cutoff point. Based on this cutoff point, we obtained the sensitivity, specificity, positive predictive value (PPV), negative predictive value (NPV), and accuracy.

Based on the developed prediction model, a nomogram consisting of the points line, predictor line, total points line, and SAP occurrence probability line was developed. In multivariate logistic regression analysis, the line of the risk factor with the largest regression coefficient was assigned 100 points, and the scores of the remaining risk factors were calculated in proportion to the largest regression coefficient. The reliability of the nomogram was verified using the area under the curve (AUC) of receiver operating characteristic (ROC) curves and calibration plots.

## 3. Results

**Figure 1:**
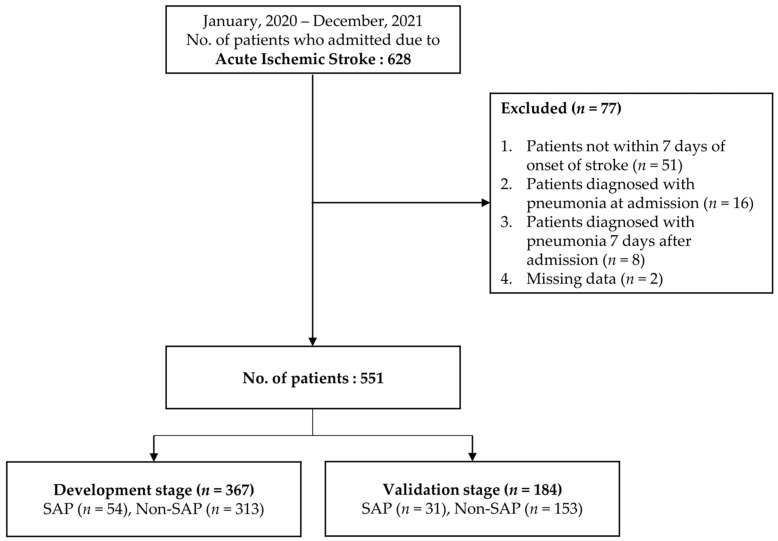
Flow chart for selection of study subjects.

We collected data from 628 subjects. Of these patients, 51 patients were not hospitalized within 7 days of stroke onset, 16 patients were diagnosed with pneumonia at the time of hospitalization, 8 patients developed pneumonia after 7 days of hospitalization, and 2 patients had insufficient data, leaving 551 patients for the study. These were randomized into 367 in the development stage and 184 in the validation stage (Figure 1).

### 3.1. Comparison of the Characteristics of SAP and Non-SAP Groups

The sociodemographic characteristics, clinical parameters, pre-existing comorbidities, and laboratory parameters of the SAP and non-SAP groups in the development stage are shown in Table 1.

The mean age of the study subjects was 71.0 years, 56.7% were male, and 17.7% of the subjects had a nasogastric tube inserted at admission.

In comparison with the non-SAP group, the SAP group had a higher proportion of subjects with an age ≥ 65 years (*p* < 0.001), an NIHSS score ≥ 7 (*p* < 0.001), dysarthria (*p* = 0.006), dysphagia (*p* < 0.001), nasogastric tube feeding (*p* < 0.001), ventilator use (*p* < 0.001), atrial fibrillation (*p* < 0.001), and congestive heart failure (*p* = 0.007). For blood tests, the SAP group had a higher proportion of all parameters out of the normal range except for blood glucose.

### 3.2. Development and Validation of the SAP Prediction Models

We conducted a multivariate logistic regression analysis of SAP incidence using factors that were found to have a significant effect on SAP incidence in univariate analysis as explanatory variables. The results showed that significant risk factors for SAP were age ≥ 65 years (OR = 3.99, *p* = 0.026), NIHSS score ≥ 7 (OR = 4.06, *p* = 0.005), nasogastric tube feeding (OR = 27.75, *p* < 0.001), and CRP ≥ 5.0 mg/dL (OR = 2.90, *p* = 0.045), and the developed logit model of SAP occurrence was E(logit of SAP) = −4.83 + 1.38 (age ≥ 65 years) + 1.40 (NIHSS score ≥ 7) + 3.32 (nasogastric tube feeding) + 1.06 (CRP ≥ 5.0 mg/dL) (Table 2). The explanatory power of the logit model was Nagelkerke R^2^ = 0.619, and the model was statistically fit with Hosmer–Lemeshow (GOF) χ^2^ = 4.424 (*p* = 0.352).

C-statistics were similar at 0.94 (95% CI 0.91–0.98, *p* < 0.001) for development and 0.91 (95% CI 0.85–0.97, *p* < 0.001) for validation. The cutoff point for maximizing sensitivity and specificity was 0.06. Based on this cutoff point, the predictive model had a sensitivity of 94.4%, specificity of 83.7%, PPV of 50.0%, NPV of 98.9%, and accuracy of 85.3% in the development stage. In the validation stage, it showed good validity with a sensitivity of 90.3%, specificity of 87.7%, PPV of 52.8%, NPV of 97.7%, and accuracy of 84.8%.

### 3.3. Development of the SAP Prediction Nomogram and Validity Evaluation

**Figure 2:**
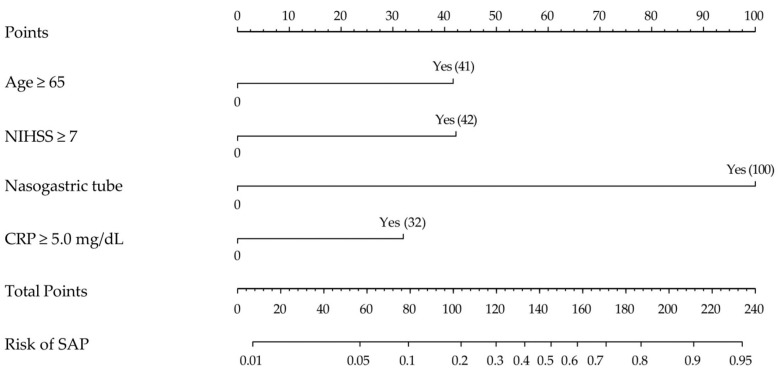
Proposed diagnostic nomogram for predicting the risk of SAP. Each predictive risk factor is given a score according to its predictive value on the point scale axis. The scores for each variable are added together to calculate the total points. Then, the total points are converted into an estimate of the probability of SAP by projecting the total points to the lower probability axis. Numbers in parentheses are points assigned if “Yes”.

We developed a nomogram based on the regression coefficients of the SAP prediction model, the logit model (Figure 2). Among the risk factors, the risk factor with the highest regression coefficient and the greatest impact was nasogastric tube feeding, which was assigned 100 points, and the remaining risk factors were assigned points in proportion to the regression coefficient of nasogastric tube feeding, such as 41 points for age ≥ 65 years, 42 points for NIHSS score ≥ 7, and 32 points for CRP ≥ 5.0 mg/dL. The sum of the scores of the risk factors in the nomogram indicated the probability of SAP.

**Figure 3:**
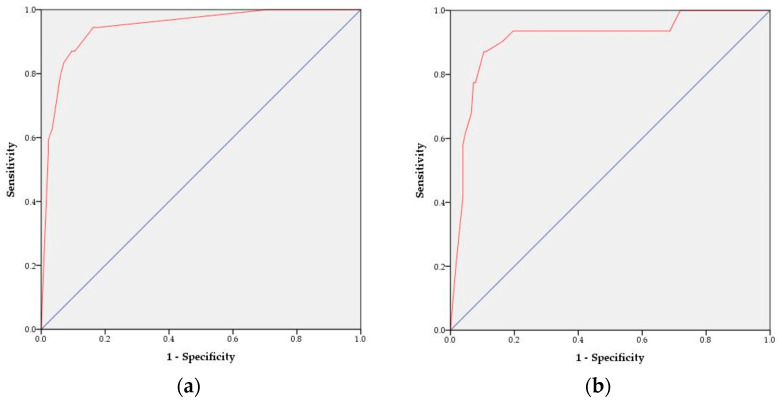
ROC curves of the SAP risk prediction nomogram: (**a**) development stage and (**b**) validation stage. The red line represents the ROC curve. The blue line represents the diagonal reference line.

**Figure 4:**
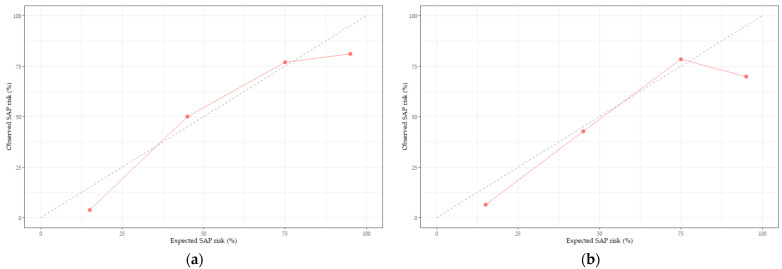
Calibration plots of the SAP risk prediction nomogram: (**a**) development stage and (**b**) validation stage. The diagonal dashed line represents the perfect calibration line. The red line represents the predicted probability versus the observed probability.

To validate the SAP prediction nomogram, we used ROC curves and calibration plots. The ROC curves of the SAP predictive nomograms developed using development and validation subjects are shown in Figure 3. The AUC value of the SAP prediction nomogram in the development stage was 0.94 (95% CI 0.91–0.98, *p* < 0.001), and the AUC value in the validation stage was 0.91 (95% CI 0.85–0.97, *p* < 0.001), which was statistically significant. Therefore, we were able to verify the efficacy of the developed nomogram. Next, we validated the nomogram using calibration plots to compare the predicted probability of SAP with the observed probability of SAP (Figure 4). The predicted probabilities calculated using the development and validation subjects were grouped based on similarity, and the slopes of the calibration plots of the development and validation subjects were close to the 45-degree line, indicating that the developed nomogram may be useful for predicting the occurrence of SAP.

## 4. Discussion

This study was conducted to predict the occurrence of SAP in clinical nursing practice through developing a SAP prediction model and a nomogram based on the risk factors of SAP occurrence in ischemic stroke patients.

The SAP rate in this study was 14.7%, which is similar to the SAP rate of around 10% reported in previous studies [21,23]. The C-statistics of this prediction model were 0.94 in the development stage and 0.91 in the validation stage, demonstrating the excellent discriminatory ability of this prediction model compared with previous prediction models (0.82–0.90) [23,28,29]. The sensitivity, specificity, PPV, NPV, and accuracy were 94.4%, 83.7%, 50.0%, 98.9%, and 85.3%, respectively. It is important to identify high-risk patients using a model that has a high sensitivity considering that SAP is associated with higher mortality rates, longer hospital stays, and higher treatment costs [30]. Previous studies have reported a sensitivity in the range of 60.5–79.3% [21,28,31,32,33]. In this study, the sensitivity was higher, thus confirming the validity of the SAP prediction model. The high discriminatory ability and sensitivity observed in this study may be explained by the classification of only pneumonia occurring within 7 days of hospitalization as SAP according to the new SAP diagnostic criteria, thus reducing the possibility of misdiagnosing hospital-acquired pneumonia as SAP. In addition, the categorization of continuous variables (risk factors) into categories based on previous studies and reference values may have contributed to the high discriminatory ability and sensitivity.

Four risk factors were identified in this study as follows: age 65 or older, NIHSS score of 7 or higher, nasogastric tube feeding, and CRP of 5.0 mg/dL or higher. Specifically, age was associated with a 3.99-fold increased risk of SAP in those aged 65 and older, consistent with previous studies reporting a higher incidence of SAP in the elderly population [29,32]. Decreased muscle strength, poor oral hygiene, and poor nutrition can increase the risk of SAP in the elderly population [34]. However, elderly people often do not have symptoms such as cough and fever even when pneumonia is present [35]; consequently, late diagnosis often worsens the disease. Therefore, it is important to identify those at risk for SAP in the elderly population and provide preventive interventions to prevent pneumonia outbreaks.

An NIHSS score of 7 or higher was associated with a 4.06 times higher risk of SAP. This result is in agreement with the findings of previous studies reporting the association of a higher NIHSS score with an increased risk of SAP [31,32,36]. The NIHSS provides an overall assessment of stroke symptoms in a short period of time. It also has the advantage of objectively assessing stroke severity, which can facilitate communication between healthcare providers about the condition of patients [37]. Nurses are the closest healthcare providers to patients, and utilizing the NIHSS in patient care can help detect stroke deterioration in an earlier stage.

In this study, nasogastric tube feeding was associated with a 27.75-fold increased risk of SAP. A previous study found that nasogastric tube feeding was associated with a 22.3-fold increase in SAP [38], which is similar to our findings. Nasogastric tube feeding is commonly used as a method of enteral feeding for patients who are unconscious or unconscious due to stroke, as well as those with dysphagia. However, SAP can be caused by aspiration due to malpositioning of the nasogastric tube [39]. To prevent this, it is necessary to perform nursing interventions such as checking the curl of the nasogastric tube in the mouth before each feeding session, examining the stomach residue through syringe suction, cleaning before and after use, and oral and nasal care. In addition, the need for a nasogastric tube should be evaluated periodically, considering the increased risk of SAP due to microaspiration with prolonged nasogastric tube feeding [3].

The percentage of subjects with nasogastric tubes in this study was 17.7%, which is higher than the nasogastric tube rate of about 8% for ischemic stroke in the United States [40]. This may be due to the higher severity of the patients in this study, which was conducted at a university hospital that is a regional emergency medical center. Therefore, multicenter studies including hospitals of varying severity are needed to generalize this nomogram to hospitals with low rates of nasogastric tube feeding.

In this study, a CRP level of 5.0 mg/dL or higher was associated with a 2.90-fold increase in SAP. This was somewhat higher in our study given that previous predictive models reported a 1.05- to 1.54-fold increase [8,41,42]. This observation may be attributed to the analysis of CRP as a continuous variable in previous studies, and a cutoff point of 5.0 mg/dL was used in this study. It is difficult to diagnose pneumonia in elderly individuals because there is usually no leukocytosis, and the chest X-ray result is mostly normal in the early stages [35]. Therefore, it is important to monitor CRP levels to predict the occurrence of pneumonia and provide prompt treatment.

Dysphagia is a condition in which there is an impediment to passing food or liquid from the mouth, through the pharynx and esophagus, and into the gastric entrance. Dysphagia has been identified as a significant risk factor in previous studies [21,31]; however, it was not included in the prediction model in this study. It was significantly associated with SAP in univariate analysis but was not statistically significant in multivariate analysis. This result may be attributed to the interaction between the variables in multivariate analysis, which could eliminate variables with relatively small effects [30]. SAP in dysphagia is most often caused by aspiration. In order to prevent aspiration pneumonia, it is important to assess the degree of swallowing and intervene early during hospitalization [3,43], and oral care is vital given that aspiration pneumonia is also caused by oropharyngeal colonization [44]. However, there is still a lack of awareness, knowledge, and skills in oral care among personnel caring for people with dysphagia. Therefore, there is a need to improve the quality of oral care through education and the implementation of oral hygiene protocols [44].

Ventilator use is also a major risk factor for pneumonia even though it was not included in the predictive model. Ventilator use in the ICU increases the risk of developing a nosocomial infection by 6 to 21 times [45]. To prevent ventilator-associated pneumonia (VAP), a VAP prevention program should be implemented, which may cover oral care, hand hygiene, care of the ventilator, ventilator circuit and ventilator accessories, patient positioning, and suction management [46].

In this study, we included the NLR and obesity as variables that were not included in the prior prediction model and found that a high NLR was associated with a 4.22-fold increase in the incidence of SAP in univariate analysis, which was not statistically significant in multivariate analysis. Although there is no established normal range for the NLR, previous studies have suggested a normal range of 0.78–3.53 [47]; thus, we used 3.53 as the upper limit of the normal range for the NLR. However, the cutoff value of the NLR has recently been suggested to be different depending on the disease [48], and further research is needed on the normal range of the NLR for stroke patients. Obesity was associated with a slightly lower risk of SAP; however, this was not statistically significant. Only one study has reported obesity as a risk factor for SAP [10], and other studies have not identified obesity as a risk factor [49]; thus, further research is needed.

In this study, we developed and validated a nomogram to easily determine the risk of SAP occurrence based on the prediction model. The AUC values were 0.94 in the development stage and 0.91 in the validation stage, showing that the discriminatory ability of the nomogram developed in this study was excellent, considering that the AUC value in a previous study was reported to be 0.85 [19]. The SAP prediction nomogram developed in this study may be utilized by nurses in clinical settings to rapidly and easily identify risk factors for SAP and screen risk groups. When caring for stroke patients, nurses should be aware of the risk factors identified in this study, such as age, NIHSS score, nasogastric tube feeding, and CRP test results, and education and active nursing interventions for nasogastric tube feeding and oral hygiene should be provided for high-risk groups. In addition, the identified risk factors for SAP should be shared with other healthcare providers to prevent the occurrence of SAP.

In some cases, nomograms are built on a website to increase their real-world clinical usability [17,50], and in the case of VAP, a study reported an increase in intervention rates and a decrease in the incidence of VAP after applying it to EMRs to check whether VAP prevention interventions were performed in real time [51]. SAP nomograms are also thought to be beneficial for SAP prevention when applied to EMRs, as they allow for continuous monitoring of records and easy verification that preventive interventions have been implemented.

In this study, we identified key risk factors for SAP in patients with acute ischemic stroke and developed a SAP prediction model and a nomogram. The methodology and results of this study are significant in that they can be used to identify patients with a high risk for SAP and facilitate early and aggressive intervention. Nasogastric tube feeding was identified as the most significant risk factor in this study. This further highlights the importance of nursing interventions for patients requiring nasogastric tube feeding, leading to more attentive nursing care, which is expected to reduce the incidence of SAP. In addition, the SAP prediction nomogram developed in this study is easy to use, allowing nurses to rapidly assess the risk of SAP. The use of the SAP prediction nomogram in actual nursing practice to promptly identify risk groups and reduce the occurrence of SAP through active nursing interventions may help maintain the mental, physical, and social health of stroke patients.

There are several limitations in this study. First, due to the retrospective study design, the data collection relied on nursing records or physician records; thus, the collected data were extremely limited, especially for smoking history and drinking history. In the future, it is necessary to plan a study that includes the specific amount and frequency of smoking and drinking in a prospective research design. Second, this study was conducted on patients diagnosed with acute ischemic stroke at a university hospital. Therefore, it is necessary to verify the validity of the findings through a multicenter replication study as it may have included a large number of patients with high severity. Third, the SAP prediction model in this study was limited by the cutoff values used for risk group classification due to the lack of prior research on appropriate reference values for stroke patients. It is necessary to identify the appropriate reference value for stroke patients through repeated studies of reference values that may affect the occurrence of complications such as SAP and mortality among stroke patients. Fourth, the age threshold in this study was 65 years old based on previous findings. Nevertheless, a follow-up study is needed to further refine the age threshold to 85 years old or older due to the recent increase in life expectancy.

## 5. Conclusions

We constructed a nomogram for SAP prediction. This study was conducted on subjects from a single university hospital, so further research on various types of medical institutions is needed for validation. Communication and cooperation among medical staff about the risk of SAP will help prevent SAP.

## Figures and Tables

**Table 1:** Comparison of the characteristics of SAP and non-SAP groups—development stage (*n* = 367).

Characteristics	Categories	Non-SAP (*n* = 313)	SAP (*n* = 54)	χ^2^/t/Z	*p*
		*n* (%)/M ± SD/Median (IQR)			
Sociodemographic Characteristics					
Age (years)	<65	104 (33.2)	5 (9.3)	12.67	<0.001
	≥65	209 (66.8)	49 (90.7)		
	M ± SD	69.83 ± 12.18	77.80 ± 9.29	−4.58	<0.001
Sex	Male	179 (57.2)	29 (53.7)	0.23	0.633
	Female	134 (42.8)	25 (46.3)		
Alcohol		89 (28.4)	9 (16.7)	3.26	0.071
Smoking		62 (19.8)	10 (18.5)	0.05	0.826
Clinical Parameters at admission					
NIHSS	<7	287 (91.7)	18 (33.3)	111.72	<0.001
	≥7	26 (8.3)	36 (66.7)		
	Median (IQR)	3.00 (1–5)	13 (5–16)	−8.11	<0.001
mRS	<3	292 (93.3)	47 (87.0)	2.56	0.110
	≥3	21 (6.7)	7 (13.0)		
	Median (IQR)	0 (0–0)	0 (0–1)	−2.24	0.025
Dysarthria		134 (42.8)	34 (63.0)	7.54	0.006
Dysphagia		25 (8.0)	44 (81.5)	162.95	<0.001
OCSP	LACS	119 (38.0)	3 (5.6)	128.24	<0.001
	TACS	21 (6.7)	36 (66.7)		
	PACS	125 (39.9)	12 (22.2)		
	POCS	48 (15.3)	3 (5.6)		
BMI	<18.5	17 (5.4)	7 (13.0)	−2.07	0.038
	18.5–22.99	114 (36.4)	26 (48.1)		
	23–24.99	73 (23.3)	8 (14.9)		
	≥25	109 (34.8)	13 (24.1)		
	M ± SD	23.64 ± 4.03	22.64 ± 4.10	1.67	0.096
Nasogastric tube		21 (6.7)	44 (81.5)	176.67	<0.001
Ventilator		2 (0.6)	10 (18.5)	46.55	<0.001
PPI (during hosplitalization)		287 (91.7)	53 (98.1)	2.81	0.093
Pre-existing Comorbidities					
Previous stroke		80 (25.6)	15 (27.8)	0.19	0.731
Hypertension		185 (59.1)	35 (64.8)	0.63	0.429
Diabetes mellitus		99 (31.6)	19 (35.2)	0.27	0.605
Chronic obstructive pulmonary disease		5 (1.6)	2 (3.7)	1.09	0.296
Atrial fibrillation		44 (14.1)	24 (44.4)	28.17	<0.001
Congestive heart failure		7 (2.2)	5 (9.3)	7.18	0.007
Ischemic heart disease		19 (6.1)	4 (7.4)	0.14	0.708
Chronic kidney disease		13 (4.2)	1 (1.9)	0.66	0.415
Laboratory Parameters at admission					
Albumin (g/dL)	≤3.5	12 (3.8)	5 (9.3)	3.07	0.008
	>3.5	301 (96.2)	49 (90.7)		
	M ± SD	4.28 ± 0.38	4.09 ± 0.51	3.24	0.001
WBC (×10^3^/uL)	<12.0	298 (95.2)	42 (77.8)	20.53	<0.001
	≥12.0	15 (4.8)	12 (22.2)		
	M ± SD	7.52 ± 2.45	9.80 ± 5.17	−5.16	<0.001
NLR	<3.53	228 (72.8)	21 (38.9)	24.34	<0.001
	≥3.53	85 (27.2)	33 (61.1)		
	M ± SD	3.03 ± 2.26	6.02 ± 5.53	−6.85	<0.001
CRP (mg/dL)	<5.0	280 (89.5)	33 (61.1)	29.49	<0.001
	≥5.0	33 (10.5)	21 (38.9)		
	M ± SD	6.42 ± 12.58	26.75 ± 63.57	−5.13	<0.001
Glucose (mg/dL)	<200	266 (85.0)	45 (83.3)	0.98	0.755
	≥200	47 (15.0)	9 (16.7)		
	M ± SD	146.94 ± 63.61	156.50 ± 87.61	−0.96	0.338
BUN (mg/dL)	<23.0	274 (87.5)	35 (64.8)	17.87	<0.001
	≥23.0	39 (12.5)	19 (35.2)		
	M ± SD	16.23 ± 7.52	19.76 ± 7.96	−3.15	0.002
Cr (mg/dL)	<1.3	280 (89.5)	40 (74.1)	9.76	0.002
	≥1.3	33 (10.5)	14 (25.9)		
	M ± SD	1.00 ± 1.07	1.01 ± 0.45	−0.06	0.953

SAP, stroke-associated pneumonia; M, mean; SD, standard deviation; IQR, interquartile range; NIHSS, National Institute of Health Stroke Scale; mRS, modified Rankin scale; OCSP, Oxfordshire Community Stroke Project; LACS, lacunar syndrome; TACS, total anterior circulation syndrome; PACS, partial anterior circulation syndrome; POCS, posterior circulation syndrome; BMI, body mass index; PPI, proton pump inhibitor; WBC, white blood cells; NLR, neutrophil-to-lymphocyte ratio; CRP, C-reactive protein; BUN, blood urea nitrogen; Cr, creatinine.

**Table 2:** Multivariate analysis of subjects—development stage (*n* = 367).

Characteristics	B Coefficient	S.E.	OR	95% CI	*p*
Constant	−4.83	0.66	0.00		<0.001
Age (years) ≥ 65	1.38	0.62	3.99	1.18–13.49	0.026
NIHSS score ≥ 7	1.40	0.50	4.06	1.54–10.72	0.005
Nasogastric tube	3.32	0.48	27.75	10.85–70.93	<0.001
CRP ≥ 5.0 (mg/dL)	1.06	0.53	2.90	1.02–8.20	0.045

Nagelkerke R^2^ = 0.619, Hosmer–Lemeshow GOF χ^2^ = 4.424, *p* = 0.352.

## Data Availability

The data presented in this study are available on request from the corresponding author.
